# Association between the persistent organic pollutants and polycystic ovary syndrome: A protocol for a systematic review and meta-analysis

**DOI:** 10.1097/MD.0000000000016948

**Published:** 2019-08-23

**Authors:** Yan Li, Mei-wei Zhang, Ying-ji Wang

**Affiliations:** a First Affiliated Hospital, Heilongjiang University of Chinese Medicine; b College of Pharmacy, Harbin Medical University, Harbin, China.

**Keywords:** meta-analysis, persistent organic pollutants, polycystic ovary syndrome, systematic review

## Abstract

**Background:**

Current evidence concerning the association between persistent organic pollutants (POPs) and polycystic ovary syndrome (PCOS) is inconsistent. The aim of the present systematic review and meta-analysis is to evaluate the role of POPs in PCOS.

**Methods:**

Databases including PubMed, Embase, Web of Science, and CNKI will be searched to identify qualified studies. All qualified studies regarding the association between POPs and PCOS will be included. The primary outcome of the present study is POPs levels in serum of subjects. Pooled analysis with corresponding 95% confidence intervals will be performed.

**Results:**

The comprehensive analysis and quantitative assessment will provide a better understanding of POPs concentrations in patients with PCOS.

**Conclusion:**

This meta-analysis and systematic review will generate evidence of the association between POPs and PCOS.

**PROSPERO registration number:**

PROSPERO CRD42019126373

## 1 Introduction

Polycystic ovary syndrome (PCOS) is the most common endocrine disorder and impacting 5% to 20% reproductive women.^[1]^ It is a complex syndrome characterized by reproductive and metabolic implications including amenorrhea/oligomenorrhea, hyperandrogenism (including hirsutism), or acne and very often by overweight and obesity.^[2,3]^

The pathogenesis of PCOS has not been fully elucidated. However, it is recognized as one of the multiple etiology diseases and seems to have close association with dysregulated steroid state, endocrine disorder, inflammatory, neuroendocrine disease, etc.^[4]^ The role of environmental factors is also under intensive investigation, and it has been demonstrated that persistent organic pollutants (POPs) are associated with PCOS, especially those could interfere with hormonal action, the so-called endocrine disrupting chemicals.^[5–8]^

The POPs are a variety of synthetic compounds, most of them are man-made^[9]^ via total synthesis. Many POPs are currently or were in the past used as pesticides, solvents, pharmaceuticals, and industrial chemicals. Since they resist photolytic, chemical, and biological degradation, POPs could hardly be degraded via biological photolytic and/or chemical process. It has been proofed that once released into the environment, POPs remain intact for exceptionally long periods of time, they accumulate in the adipose tissue of living organisms including humans.^[9,10]^

The POPs play an important role in the development of a variety of diseases because they have toxic, carcinogenic, and endocrine-disrupting properties that either mimic or block endogenous hormones and thus disrupt normal hormone homeostasis.^[11,12]^

Till now, POPs are recognized as risk factors in the pathogenesis of breast cancer,^[13]^ prostate cancer,^[14]^ thyroid cancer,^[15]^ obesity, type 2 diabetes,^[16,17]^ and hypertension.^[18]^ It is also demonstrated that POPs can damage reproductive and developmental systems of human being, and are associated with risks of female reproductive disorders such as primary ovarian insufficiency,^[19,20]^ early menopause,^[21]^ younger ages of attaining menarche and early sexual maturation,^[22,23]^ prolonged time to pregnancy,^[24]^ pregnancy loss,^[25,26]^ and PCOS.^[5–8]^

The POPs is a variety of synthetic compounds, although studies have reported the association between certain types of POPs and the risk of PCOS, the current evidence are inconsistent due to the small sample size of designs and the chemical properties. Therefore, it is of great importance to perform a systematic review and meta-analysis to target high risk POPs, it will facilitate a better understanding of what kind of POPs are involved in the pathogenesis of PCOS and provide suggestions for avoiding exposure in daily behavior.

## 2 Methods and analysis

The review team develops the methods for the present systematic review and meta-analysis following the PRISMA statement for preferred reporting items for systematic reviews^[27]^ and meta-analysis protocols and the Meta-analysis Of Observational Studies in Epidemiology (MOOSE) guidelines.^[28]^

### 2.1 Eligibility criteria

#### 2.1.1 Types of studies

The present systematic review will include published articles if they met all of the following criteria: original observational studies on human participants published; exposure related to POPs; outcome of interest is PCOS; studies reported POPs levels in females with PCOS and healthy controls; studies provided POPs means and standard deviation (SD) or provide enough information to calculate such a measure. We will exclude reviews, meta-analyses, ecological studies, case series, case reports, policy papers, and comments.

#### 2.1.2 Types of population

The review will include patients with PCOS diagnosed with revised Rotterdam Criteria 2003,^[29]^ National Institute of Health (NIH) 1990 criteria,^[30]^ or Androgen Excess and Polycystic Ovary Syndrome (AE-PCOS) Society criteria.^[31]^

#### 2.1.3 Types of exposures

The exposure of interest will be the POPs including polychlorinated biphenyls, polycyclic aromatic hydrocarbons, *p*,*p*′-dichlorodiphenyldichloroethylene, etc.

#### 2.1.4 Types of outcome measures

The primary outcome is to identify types of POPs and their levels in serum of subjects.

### 2.2 Search strategy

**Figure 1:**
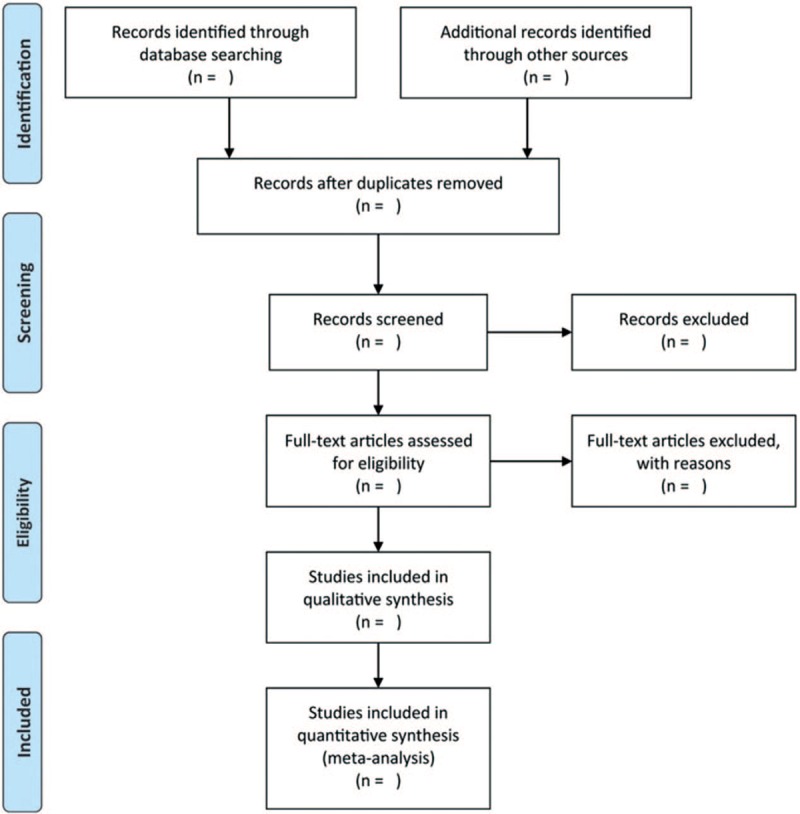
Flowchart of study selection.

The reviewers will conduct a systematic literature search in the following electronic databases: PubMed, Embase, Web of Science, and CNKI without language limitations. The search dates will be set from the inception to June 2019. MeSH words and free words with the following searching strategy will be used: (“persistent organic pollutants” OR “pesticide” OR “polychlorinated biphenyls” OR “dichlorodiphenyltrichloroethane” OR “polycyclic aromatic hydrocarbons”) AND (“polycystic ovary syndrome” OR “Stein-Leventhal syndrome” OR “polycystic ovarian syndrome” OR “polycystic ovarian disease”). We will also manually retrieve conference proceedings and academic exchange summaries. The whole process of study selection is summarized as flowchart in Figure 1.

### 2.3 Data extraction and management

Two independent investigators (YL and MZ) will extract and tabulated all data using a standardized data extraction form. Discrepancies will be resolved via referencing the original article and via group discussions or in consultation with the principal investigator (YW). The following data will be extracted leading author, year of publication, journal, country or region, study design, sample size, diagnostic criteria of PCOS, number of PCOS cases, POPs type, POPs concentrations, relative risks of PCOS comparing various levels of POPs, risk estimate confounders, and main conclusion. Investigators will contact the corresponding author of each included study for further information if any important data are missing.

### 2.4 Appraisal of quality and risk bias

Quality of included studies will be assessed using the Newcastle–Ottawa Scale.^[32]^ The NOS assessment consisted of 3 major categories: selection (1 star for each terms), comparability (up to 2 stars), and exposure (1 star for each terms). The score is positively associated with the quality of the study. Funnel plot will be implemented to detect the risk of publication bias if there are more than 10 studies qualified for analysis. Otherwise, Begg test and Egger test will be used.

### 2.5 Data synthesis

The POPs concentrations will be presented as mean and SD. SD value will be transformed if only standard error or the 95% confidence interval is provided. If only median and range are available, mean and SD will be transformed following previously described forum: Mean ≈ Median; SD ≈ Norm IQR = (P_75_ − P_25_) × 0.7413 (IQR = interquartile range, P_75_ = 75th percentile, P_25_ = 25th percentile).^[33]^

Heterogeneity across enrolled studies will be quantified using the *Q*-statistic and inconsistency index (*I*^2^).^[34]^*I*^2^ > 50%, heterogeneity will be considered as severe; if *I*^2^ = 25% to 50%, heterogeneity will be considered as moderate, and if *I*^2^ < 25%, heterogeneity will be considered as low. A random-effects model will be used when it is severe heterogeneity, otherwise, a fixed-effect model will be applied. All analyses will be carried out in STATA software (Version 14.0; Stata Corporation, College Station, TX).

### 2.6 Ethics and dissemination

Since this is a systematic review and meta-analysis, ethics approval is not required.

We will report our findings of this systematic review and meta-analysis in a peer-reviewed journal in the future.

## Author contributions

**Conceptualization:** Yan Li, Yingji Wang.

**Data curation:** Yan Li, Meiwei Zhang, Yingji Wang.

**Formal analysis:** Meiwei Zhang, Yingji Wang.

**Funding acquisition:** Yan Li, Yingji Wang.

**Investigation:** Yan Li, Meiwei Zhang, Yingji Wang.

**Methodology:** Yan Li, Meiwei Zhang, Yingji Wang.

**Project administration:** Yan Li.

**Software:** Meiwei Zhang, Yingji Wang.

**Supervision:** Yan Li.

**Validation:** Yan Li.

**Writing – original draft:** Yan Li, Meiwei Zhang, Yingji Wang.

**Writing – review & editing:** Yan Li, Meiwei Zhang, Yingji Wang.

## References

[1] Azziz R Carmina E Chen Z Polycystic ovary syndrome. Nat Rev Dis Primers 2016;11:16057. 10.1038/nrdp.2016.57 27510637

[2] Ehrmann DA Polycystic ovary syndrome. N Engl J Med 2005;352:1223–36. 15788499 10.1056/NEJMra041536

[3] Orio F Palomba S Reproductive endocrinology: new guidelines for the diagnosis and treatment of PCOS. Nat Rev Endocrinol 2014;10:130–2. 24322653 10.1038/nrendo.2013.248

[4] Li Y Chen C Ma Y Multi-system reproductive metabolic disorder: significance for the pathogenesis and therapy of polycystic ovary syndrome (PCOS). Life Sci 2019;228:167–75. 31029778 10.1016/j.lfs.2019.04.046

[5] Yang Q Zhao Y Qiu X Association of serum levels of typical organic pollutants with polycystic ovary syndrome (PCOS): a case-control study. Hum Reprod 2015;30:1964–73. 26040477 10.1093/humrep/dev123

[6] Guo Z Qiu H Wang L Association of serum organochlorine pesticides concentrations with reproductive hormone levels and polycystic ovary syndrome in a Chinese population. Chemosphere 2017;171:595–600. 28043072 10.1016/j.chemosphere.2016.12.127

[7] Vagi SJ Azziz-Baumgartner E Sjödin A Exploring the potential association between brominated diphenyl ethers, polychlorinated biphenyls, organochlorine pesticides, perfluorinated compounds, phthalates, and bisphenol A in polycystic ovary syndrome: a case-control study. BMC Endocr Disord 2014;14:86. 25348326 10.1186/1472-6823-14-86 PMC4287339

[8] Heffernan AL Cunningham TK Drage DS Perfluorinated alkyl acids in the serum and follicular fluid of UK women with and without polycystic ovarian syndrome undergoing fertility treatment and associations with hormonal and metabolic parameters. Int J Hyg Environ Health 2018;22:1068–75. 10.1016/j.ijheh.2018.07.009 30037723

[9] El-Shahawi MS Hamza A Bashammakhb AS An overview on the accumulation, distribution, transformations, toxicity and analytical methods for the monitoring of persistent organic pollutants. Talanta 2010;80:1587–97. 20152382 10.1016/j.talanta.2009.09.055

[10] Manahan S Environmental Chemistry. 7th ed London: Lewis Publishers/CRC Press LLC; 2000.

[11] Mnif W Hassine AI Bouaziz A Effect of endocrine disruptor pesticides: a review. Int J Environ Res Public Health 2011;8:2265–303. 21776230 10.3390/ijerph8062265 PMC3138025

[12] Bonefeld-Jorgensen EC Ghisari M Wielsoe M Biomonitoring and hormone-disrupting effect biomarkers of persistent organic pollutants in vitro and ex vivo. Basic Clin Pharmacol Toxicol 2014;115:118–28. 24797035 10.1111/bcpt.12263 PMC4270256

[13] Mouly TA Toms LL Breast cancer and persistent organic pollutants (excluding DDT): a systematic literature review. Environ Sci Pollut Res Int 2016;23:22385–407. 27628920 10.1007/s11356-016-7577-1

[14] Lim JE Park SH Jee SH Body concentrations of persistent organic pollutants and prostate cancer: a meta-analysis. Environ Sci Pollut Res Int 2015;22:11275–84. 25797015 10.1007/s11356-015-4315-z

[15] Han MA Kim JH Song HS Persistent organic pollutants, pesticides, and the risk of thyroid cancer: systematic review and meta-analysis. Eur J Cancer Prev 2019;28:334–49. 10.1097/CEJ.0000000000000481 30362975

[16] Yang C Kong APS Cai Z Persistent organic pollutants as risk factors for obesity and diabetes. Curr Diab Rep 2017;17:132. 29098478 10.1007/s11892-017-0966-0

[17] Henríquez-Hernández LA Luzardo OP Valerón PF Persistent organic pollutants and risk of diabetes and obesity on healthy adults: Results from a cross-sectional study in Spain. Sci Total Environ 2017 607–8. 28724247 10.1016/j.scitotenv.2017.07.075

[18] Park SH Lim JE Park H Body burden of persistent organic pollutants on hypertension: a meta-analysis. Environ Sci Pollut Res Int 2016;23:14284–93. 27055888 10.1007/s11356-016-6568-6

[19] Pan W Ye X Yin S Selected persistent organic pollutants associated with the risk of primary ovarian insufficiency in women. Environ Int 2019;129:51–8. 31108393 10.1016/j.envint.2019.05.023

[20] Li C Cao M Ma L Pyrethroid pesticide exposure and risk of primary ovarian insufficiency in Chinese women. Environ Sci Technol 2018;52:3240–8. 29444570 10.1021/acs.est.7b06689

[21] Grindler NM Allsworth JE Macones GA Persistent organic pollutants and early menopause in U.S. women. PLoS One 2015;10:e0116057. 25629726 10.1371/journal.pone.0116057 PMC4309567

[22] Denham M Schell LM Deane G Relationship of lead, mercury, mirex, dichlorodiphenyldichloroethylene, hexachlorobenzene, and polychlorinated biphenyls to timing of menarche among Akwesasne Mohawk girls. Pediatrics 2005;115:e127–34. 15653789 10.1542/peds.2004-1161

[23] Rogan WJ Ragan NB Evidence of effects of environmental chemicals on the endocrine system in children. Pediatrics 2003;112:247–52. 12837917

[24] Yang CY Wang YJ Chen PC Exposure to a mixture of polychlorinated biphenyls and polychlorinated dibenzofurans resulted in a prolonged time to pregnancy in women. Environ Health Perspect 2008;116:599–604. 18470317 10.1289/ehp.10715 PMC2367681

[25] Longnecker MP Klebanoff MA Dunson DB Maternal serum level of the DDT metabolite DDE in relation to fetal loss in previous pregnancies. Environ Res 2005;97:127–33. 15533328 10.1016/S0013-9351(03)00108-7

[26] Tsukimori K Tokunaga S Shibata S Long-term effects of polychlorinated biphenyls and dioxins on pregnancy outcomes in women affected by the Yusho incident. Environ Health Perspect 2008;116:626–30. 18470296 10.1289/ehp.10686 PMC2367658

[27] Shamseer L Moher D Clarke M Preferred reporting items for systematic review and meta-analysis protocols (PRISMA-P) 2015: elaboration and explanation. BMJ 2015;350:g7647. 25555855 10.1136/bmj.g7647

[28] Stroup DF Berlin JA Morton SC Meta-analysis of observational studies in epidemiology: a proposal for reporting. Meta-analysis Of Observational Studies in Epidemiology (MOOSE) group. JAMA 2000;283:2008–12. 10789670 10.1001/jama.283.15.2008

[29] Rotterdam ESHRE/ASRM--Sponsored PCOS Concensus Workshop Group. Revised 2003 consensus on diagnostic criteria and long-term health risks related to polycystic ovary syndrome (PCOS). Hum Reprod 2004;19:41–7. 14688154 10.1093/humrep/deh098

[30] Zawadski JK Dunaif A Diagnostic criteria for polycystic ovary syndrome: towards a rational approach. In: Dunaif A Givens JR Haseltine F, eds. Polycystic Ovary Syndrome. Vol. 1992. Boston, MA: Blackwell Scientific 1992 pp. 377–384.

[31] Azziz R Carmina E Dewailly D Positions statement: criteria for defining polycystic ovary syndrome as a predominantly hyperandrogenic syndrome: an Androgen Excess Society guideline. J Clin Endocrinol Metab 2006;91:4237–45. 16940456 10.1210/jc.2006-0178

[32] Stang A Critical evaluation of the Newcastle-Ottawa scale for the assessment of the quality of nonrandomized studies in meta-analyses. Eur J Epidemiol 2010;25:603–5. 20652370 10.1007/s10654-010-9491-z

[33] Yang L Wang G Du Y Remote ischemic preconditioning reduces cardiac troponin I release in cardiac surgery: a meta-analysis. J Cardiothorac Vasc Anesth 2014;28:682–9. 24103716 10.1053/j.jvca.2013.05.035

[34] Higgins JP Thompson SG Deeks JJ Measuring inconsistency in meta-analyses. BMJ 2003;327:557–60. 12958120 10.1136/bmj.327.7414.557 PMC192859

